# Do religious people in Western Balkans have faith in COVID-19 vaccines? Smiljana Cvjetkovic

**DOI:** 10.1093/eurpub/ckac131.359

**Published:** 2022-10-25

**Authors:** S Cvjetkovic, V Jeremic Stojkovic, J Jankovic, S Mandic-Rajcevic

**Affiliations:** 1 Department of Humanities, Faculty of Medicine, University of Belgrade, Belgrade, Serbia; 2 Institute of Social Medicine, Faculty of Medicine, University of Belgrade, Belgrade, Serbia

## Abstract

**Background:**

Recently, it was established that more religious people tend to have less favorable views of vaccination. The aim of this study was to assess the relationship between the religiousness and attitudes towards COVID-19 vaccination and disease, in five Western Balkans countries.

**Methods:**

Using online questionnaire and convenience sampling procedure, data were obtained from 1605 respondents aged 18-75 years. Perceived COVID-19 vaccine safety and efficacy, and observed danger and susceptibility to disease were assessed by short five-point Likert scales. Religiousness was estimated using single item scale. Multivariate regression analysis was employed.

**Results:**

In Serbia, respondents who assessed themselves as more religious considered vaccine against COVID-19 as less safe (β=-.10, p<.01) and effective (β=-.12, p<.01), were more prone to the attitude that dangers of COVID-19 are not that serious (β=-.09, p<.01), and believed to a greater extent that they are less susceptible to the virus (β=-.07, p<.01). Similarly, in Bosnia and Herzegovina more religious individuals were less convinced that vaccine is effective (β=-.07, p<.05), less inclined to believe that danger of the virus is serious (β=-.07, p<.05), and assessed their susceptibility as lower (β=-.06, p<.05). More religious people in Montenegro (β=-.06, p<.05) regarded the vaccine as less safe, while in North Macedonia (β=.06, p<.05) and Albania (β=.08, p<.01) stronger religiosity was associated with more favorable attitudes towards vaccine safety.

**Conclusions:**

The relationship between religiosity and attitudes towards COVID-19 vaccination and disease is culturally conditioned. While in some Western Balkans societies religious beliefs render individual with a sense of lack of control triggering worry and anxiety, in some others they work as psychological shield against existential threats.

**Key messages:**

• Religiosity should be considered as a relevant factor in vaccination campaigns implementation.

• In the societies where negative attitudes towards vaccination prevail among the believers, religious leaders should be peculiarly educated and encouraged to participate in vaccination campaigns.

